# Effect of Multi-Modal Therapies for Kinesiophobia Caused by Musculoskeletal Disorders: A Systematic Review and Meta-Analysis

**DOI:** 10.3390/ijerph17249439

**Published:** 2020-12-16

**Authors:** Yining Xu, Yang Song, Dong Sun, Gusztáv Fekete, Yaodong Gu

**Affiliations:** 1 Faculty of Sports Science, Ningbo University, Ningbo 315211, China; xuyining_nbu@foxmail.com; 2 Doctoral School of Safety and Security Sciences, Obuda University, 1034 Budapest, Hungary; nbusongyang@hotmail.com; 3 Faculty of Engineering, University of Szeged, 6724 Szeged, Hungary; 4 Savaria Institute of Technology, Eötvös Loránd University, 9700 Szombathely, Hungary; fg@inf.elte.hu

**Keywords:** kinesiophobia, pain, multi-modal, psychological measure, Tampa scale

## Abstract

This systematic review and meta-analysis aimed to identify the effect of multi-modal therapies that combined physical and psychological therapies for kinesiophobia caused by musculoskeletal disorders compared with uni-modal therapy of only phycological therapy or psychological therapy. The search terms and their logical connector were as following: (1) “kinesiophobia” at the title or abstract; and (2) “randomized” OR “randomized” at title or abstract; not (3) ”design” OR “protocol” at the title. They were typed into the databases of Medline (EBSCO), PubMed, and Ovid, following the different input rules of these databases. The eligibility criteria were: (1) Adults with musculoskeletal disorders or illness as patients; (2) Multi-modal therapies combined physical and psychological therapy as interventions; (3) Uni-modal therapy of only physical or psychological therapy as a comparison; (4) The scores of the 17-items version of the Tampa Scale of Kinesiophobia as the outcome; (5) Randomized controlled trials as study design. As a result, 12 studies were included with a statistically significant polled effect of 6.99 (95% CI 4.59 to 9.38). Despite a large heterogeneity within studies, multi-modal therapies might be more effective in reducing kinesiophobia than the unimodal of only physical or psychological therapy both in the total and subdivision analysis. The effect might decrease with age. What’s more, this review’s mathematical methods were feasible by taking test-retest reliability of the Tampa Scale of Kinesiophobia into consideration.

## 1. Introduction

Musculoskeletal disorders and the following pain were common in most people’s daily life [1], and the musculoskeletal pain caused by musculoskeletal disorders was the second most common cause of disability [2]. Many established factors, such as physical, biological, cognitive, behavioral, social, and occupation, were correlated with the pain following musculoskeletal disorders [1,3,4].

Fear was considered to be an explanation of why pain and associated outcomes such as disability persist once the body injury had healed [5,6], and the fear-avoidance model of pain was one of the frameworks which could explain the development and persistence of the pain and disability following a musculoskeletal injury [7,8]. According to this model, people with a trait tend to have fear and catastrophic thoughts in response to pain were more at risk of developing chronic musculoskeletal pain after an injury than people who did not have this tendency [6,8]. These people over-reacted in response to actual or potential threats, developing avoidance behaviors to prevent a new injury/re-injury [6]. Fear in relation to pain had been described with various conceptual definitions, among which pain-related fear, fear-avoidance beliefs, fear of movement, and kinesiophobia were the most commonly used [9].

Kinesiophobia was one of the most commonly used conceptual definitions which could describe fear in relation to pain [10]. Kinesiophobia (also known as the fear of movement) was defined as an excessive, irrational, and debilitating fear to carry out a physical movement due to a feeling of vulnerability to a painful injury or re-injury [10]. It could be acquired through a direct aversive experience such as pain and trauma or through social learning such as observation and instruction [11]. Kinesiophobia had been associated with pain, disability, and quality of daily life to some extent [12]. The prevalence of kinesiophobia in chronic pain was from 50% to 70% [13,14].

The objective of rehabilitation is to recover physical exercises’ performance, regain the capacity of daily activities, and restore social functions. In recent years, studies on the rehabilitation of musculoskeletal disorders had begun to focus on the fear in relation to pain [15,16]. The fear in relation to pain would cause people to produce fear-avoidance and had a negative effect on their quality of life. Therefore, not only the rehabilitation at the physical level but also the rehabilitation at the psychological level should be paid attention to [17,18,19,20]. At the same time, as mentioned above, kinesiophobia could be acquired through many different ways (e.g., personal experience, social learning) [11], therapies combined multi-modal from both psychological and physical perspectives had become increasingly popular [15,21,22]. However, at present, only a few studies focus on the advantage of multi-modal therapies over uni-modal therapies, and most of the studies on the rehabilitation of musculoskeletal disorders were limited to specific musculoskeletal disorders. There was a lack of high-quality evidence from the macro-perspective. To answer this question, the terms “physical therapy” and “psychological therapy” should be defined in this review at first.

In this review, the terms “physical therapy” and “psychological therapy” were defined as follows. “Physical therapy” was the therapy included: (1) exercise/training session or advice with a private plan; (2) passive physical therapies such as usual care; (3) treatments provided by professional therapists or medical staff without any psychological education. It should be emphasized that exercise/training advice without a private plan, waiting lists and interventions without any control, such as keeping normal daily life, would be excluded.

“Psychological therapy” was the therapy include (1) psychological education; (2) cognition-behavior therapy; (3) perceptive stimulation in non-injured body areas such as virtual reality equipment, laser, and relaxation; (4) therapeutic milieu involves interpersonal communication such as group session and feedback session. It should be emphasized that if the doctor-patient communication in the intervention involved only an explanation of the treatment or only guidance of exercise or only supervision in training, the intervention wouldn’t be regarded as psychological therapy.

Moreover, a quantitative indicator was required to assess the fear of movement, and there was not a specific tool to assess fear of movement directly [9]. The term “kinesiophobia” would be used. People with kinesiophobia would change their movements to avoid pain and adjust their motor behaviors [12]. The processing of pain and pain-related information in people with musculoskeletal disorders could be related to how kinesiophobia was perceived [23]. Therefore, a greater degree of kinesiophobia predicted greater levels of fear of pain and a great inclination to avoid physical movements [24].

Kinesiophobia could be measured by the Tampa Scale of Kinesiophobia (TSK) [25]. Since the Tampa Scale of Kinesiophobia had good validity in the quantization of kinesiophobia, the change of TSK scores would reflect the effect of therapy and be taken as a comparative indicator of therapy effect to some extent [26,27]. In the original version of the Tampa Scale of Kinesiophobia, participants would be asked to respond to how much they agreed with each of the 17 items, and the ratings available were: (1) disagree; (2) partially disagree; (3) partially agree; (4) strongly agree. The score of each item varies from 1–4 or 0–3. The responses were summed, and the generated score, which ranges from 17 to 68 or from 0 to 51 [13,25]. However, the TSK scores were usually reported as a secondary outcome, and that there was a limited number of studies that focus on the treatments for kinesiophobia. It made that a special study search strategy, information extraction, and data processing methods need to be applied.

This systematic review and meta-analysis aimed to identify the effect of multi-modal therapies, which combined physical and psychological therapies following the definition mentioned above, for kinesiphobia caused by musculoskeletal disorders compared with uni-modal therapies of an only physical or only psychological therapy that followed the definitions mentioned above.

## 2. Methods

### 2.1. Protocol and Registration

This review was conducted in accordance with the Preferred Reporting Items for Systematic Reviews and Meta-Analyses (PRISMA) statement (Registration number: CRD42020218384) [28].

### 2.2. Eligibility and Exclusion Criteria And Rationale of PICOS

Studies were included in this review if they met the following eligibility criteria: (1) Patients should be adults with musculoskeletal disorders or illness. What should be emphasized was that the patients with the injuries and their following pain, which cause by surgery and other diseases of tissues and organs, would be excluded; (2) Intervention should be multi-modal therapy, which combined physical and psychological therapy. The definitions of physical therapy and psychological therapy were given in the introduction. For example, a cognitive-behavioral group intervention contained a skill training plan would be regarded as multi-modal therapy; (3) The comparison was multi-modal therapies versus uni-modal therapies. The uni-modal therapy should be physical therapy or psychological therapy, the definitions of which were given in the introduction. For example, a supervised home exercise with a given plan would be regarded as a uni-modal therapy from a physical perspective; (4) Outcomes would be scores of the 17-items version of the Tampa Scale of Kinesiophobia; (5) Randomized controlled trials; (6) Date and Language: published in English from inception to September 2020.

The 17-items Tampa Scale of Kinesiophobia had been demonstrated predictive and constructed validity as well as excellent test-retest reliability (0.64–0.91) and internal consistency (0.70–0.81). What should be paid attention to was that there were some shorten versions of the Tampa Scale of Kinesiophobia, such as TSK-11, TSK-13, and the Tampa Scale of Kinesiophobia for particular injuries. All of these versions demonstrated high validities, reliabilities, and consistencies as well. To ensure the consistency of results and reduce the heterogeneity, only the 17-items version would be chosen as this review’s outcome, and the 17-items version whose scores ranged from 0 to 3 would be converted to 1 to 4.

Studies would be excluded if they met the following eligibility criteria: (1) Healthy people, children, and teenagers; (2) pain caused by trauma, burns, or surgery; (3) other versions of the Tampa Scale of Kinesiophobia; (4) lack of origin scores of the Tampa Scale of Kinesiophobia.

### 2.3. Search and Study Selection

The search terms and their logical connector were as following: (1) “kinesiophobia” at the title or abstract; and (2) “randomized” OR “randomized” at title or abstract; not (3) “design” OR “protocol” at the title. They were typed into the databases of Medline (EBSCO), PubMed, and Ovid, following the different input rules of these databases.

In the database PubMed, the search term”(kinesiophobia[Title/Abstract]) AND ((randomized) or (randomised)[Title/Abstract]) NOT ((design) or (protocol)[Title])” was used, then the results would be limited at “randomized controlled trials”. In the database Medline (EBSCO), the search term “AB kinesiophobia and AB (randomized or randomised) NOT TI (design or protocol)” was used, and the range of search would be fixed in Cochrane Central Register of Trials, Medline with Full Text, and CINAHL. In the database Ovid, “All Resources” would be chosen, and the search term “(kinesiophobia and (randomized or randomised) .ab not (design or protocol) .at” was used with the tool “Multi-Field Search”, and the language would be limited in English. All the results would be downloaded and imported into EndNote X9 for further screening.

With the help of the functions “deduplication” and “searching in library” of EndNote X9, the duplicated and ineligible studied would be screened.

A unified method and standard of study search and Selection were used, in which the two authors (Y.X. and Y.S.) searched the study in turn, independently. The figures and tables for information would be made after the two authors verified their results finally. An independent arbitrator (D.S) resolved any discrepancies in data extraction.

### 2.4. Data Extraction and Management

A unified method and standard of data extraction were used, in which the two authors (Y.X. and Y.S.) extracted the information in turn, independently. The figures and tables for information would be made after the two authors verified their data finally. An independent arbitrator (D.S.) resolved any discrepancies in data extraction.

The following data are collected. (1) The versions of The Tampa Scale of Kinesiophobia and their test-retest reliabilities, which could be searched in the references of these studies, or be inferred from the nationalities of the participants; (2) The origin scores of the Tampa Scale of Kinesiophobia at baselines and different follow-up times; (3) The mean age of participants which would be combined by statistical Formulas; (4) The types of musculoskeletal pain; (5) The duration of interventions.

All the scores of the Tampa Scale of Kinesiophobia would be converted to the form of MEAN (SD). If an included study reported the Tampa Scale of Kinesiophobia scores of different follow-up times or had more than one trial arm, each different follow-up times and trial arm would be treated as a separate trial [29].

It should be paid attention that some original data of included studies should be revised to unify and standardize. The corrected mean differences and standard deviations of the Tampa Scale of Kinesiophobia scores of the baselines and the follow-up times could be calculated by the basic statistical Formulas (1) and (2) (The number 1 means the baseline and the number 2 represents the follow-ups). The correlation coefficient R was the test-retest reliability or the internal consistency coefficient of the Tampa Scale of Kinesiophobia, which was used in the included studies. Different language versions of the Tampa Scale had different test-retest reliabilities. If a study didn’t report the version of the Tampa Scale of Kinesiophobia, the rest-retest reliability would be inferred from the participants’ nationalities or references of the study:

(1) $$MD=MD_{1}-MD_{2},$$

(2) $$SD=\sqrt{SD_{1}^{2}+SD_{2}^{2}-2R\cdot SD_{1\cdot }SD_{2}},$$

Since the scores of the Tampa Scale of Kinesiophobia were in the same units, the standardized mean difference was not chosen to illustrate the pooled effect. The software RevMan5.3 was used to analyze the combined effect, and the random effect model (REM) was chosen. All the total and subtotal results would be shown in the forest plots made by RevMan5.3.

The size of heterogeneity would be tested by two indexes, one of which was I^2^, a qualitative and descriptive index showing the inconsistency of the included studies, and the other was Tau^2^, an quantitative and analytical index showing the true difference. The statistic Q, which was equal to the statistic Chi^2^ calculated by the RevMan5.3, and the confidence intervals of T^2^ and I^2^, could explain whether the result has the true heterogeneity.

The prediction intervals of all the statistics mentioned above, which combines the estimation of the mean effect with the variance of the true effect and approximate the actual dispersion of the true effect as the number of studies tend to infinity, were calculated as well.

### 2.5. Risk of Bias and Quality of Evidence

A unified assessment method and standard of the risk of bias were used, in which the two authors (Y.X. and Y.S.) assessed each study in turn, independently. The figures for information would be made after the two authors verified their data finally. An independent arbitrator (D.S.) resolved any discrepancies in the assessment of the risk of bias. The Cochrane Collaboration’s tool was used to assess the risk of bias [30]. The trials’ quality was assessed and graded independently by two authors according to the criteria described in The Cochrane Handbook. And the risk of bias graph and the risk of bias summary graph would be made by RevMan5.3.

A Funnel plot based on the calculation of standard mean difference (SMD) was used in this review to describe the existence of bias across studies. The analysis of bias risk was based on the following model assumptions. (1) Studies with large sample sizes were more likely to be published; (2) Studies on medium-sized samples were easily omitted; (3) Studies with small sample sizes were most likely to be missed.

The Grading of Recommendations Assessment, Development, and Evaluation (GRADE) system, which would be made by the software GRADEprofiler (Version 3.2.2), was used to illustrate the quality of evidence [31].

### 2.6. Addition Analysis

#### 2.6.1. Subgroup Analysis

If the heterogeneity of the pooled effect were large, a subgroup analysis would be necessary to evaluate the source of heterogeneity and the influencing factors of the effect. The heterogeneity between the studies might come from different types of musculoskeletal pain, different follow-up times, and the different mean age of the participants, and different durations of treatments. Therefore, the subgroup analysis would be conducted from the following aspects: (1) Types of musculoskeletal pain; (2) Follow-up times; (3) Mean of the participants; (4) Duration of Treatments; (5) Different control group interventions. All the continuous variables would be divided into several subgroups for analysis. However, the subgroup analysis might not explain the source of heterogeneity. For a certain covariate, a further meta-regression would be made if there were enough studies.

#### 2.6.2. Meta-Regression

Meta-Regression, whose results could explain the correlation of different subgroup deviations and the pooled effect, would be made by the software STATA^®^ 14. Following the meta-regression principles, the restricted maximum likelihood (REML) was used in meta-regression, and the regression coefficient is expressed in exponential form (exp). Moreover, the Monte Carlo Method was used to correct the P-value to verify the existence of the type I error. The times of calculation was set to 5000.

#### 2.6.3. Statistical Power

Generally, the statistical power should be analyzed before primary studies or a meta-analysis. However, to verify the rationality of this review’s mathematical method, the statistical power of every primary study, meta-analysis, and heterogeneity analysis would be calculated. Generally, although researchers often wanted the statistical power of their study to be at least 80%, a single primary study’s statistical power was usually low. The statistical power of studies from medicine, psychology, education, and other fields of science were generally less than 80% and less than 50% when testing for low effects. Since a meta-analysis had a larger sample size than any included study, it usually had higher statistical power. In this review, each included studies’ statistical power, the meta-analysis, the subgroup analysis, and the heterogeneity test were calculated.

## 3. Results

### 3.1. Study Selection, Information, and Original Data

In the database PubMed, by the search term “(kinesiophobia[Title/Abstract]) AND ((randomized) or (randomised)[Title/Abstract]) NOT ((design) or (protocol)[Title])”, and the limitation of “randomized controlled trials”, 55 studies had been screened out. In the database Medline (EBSCO), by the search term “AB kinesiophobia and AB (randomized or randomised) NOT TI (design or protocol)”, and the range of “Cochrane Central Register of Trials, Medline with Full Text, and CINAHL”, 507 studies had been screened out. In the database Ovid, by the search term “(kinesiophobia and (randomized or randomised) .ab not (design or protocol) .at” typed in the tool “Multi-Field Search”, and the limitation of English, 480 studies had been screened out. All the results would be downloaded and imported into EndNote X9 for further screening.

**Figure 1:**
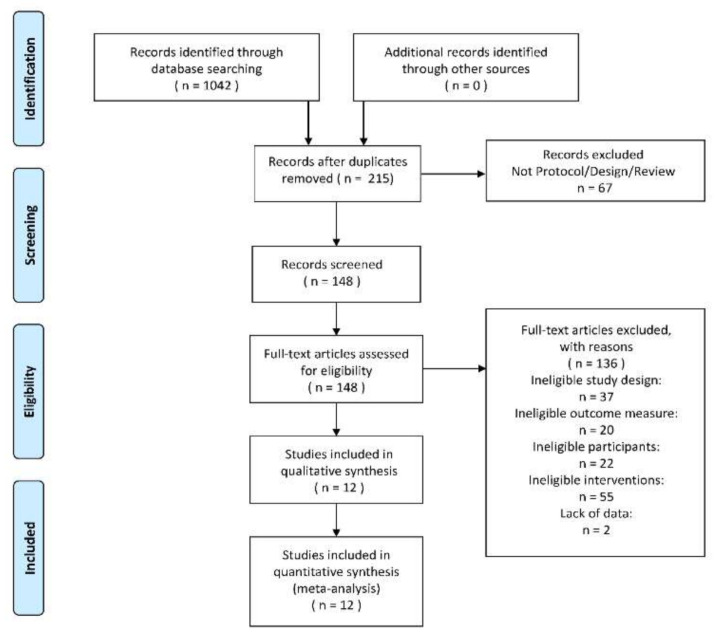
The PRISMA 2009 flow diagram of search and study selection.

After deduplication and applying the exclusion criteria, 12 studies [32,33,34,35,36,37,38,39,40,41,42,43] of the total 1042 studies were included for analysis. The flow diagram could be seen in Figure 1. Based on the information of all the full texts included, the result of data collection and a summary measure of each included study could be seen in Table 1 and Table 2.

### 3.2. Risk of Bias

**Figure 2:**
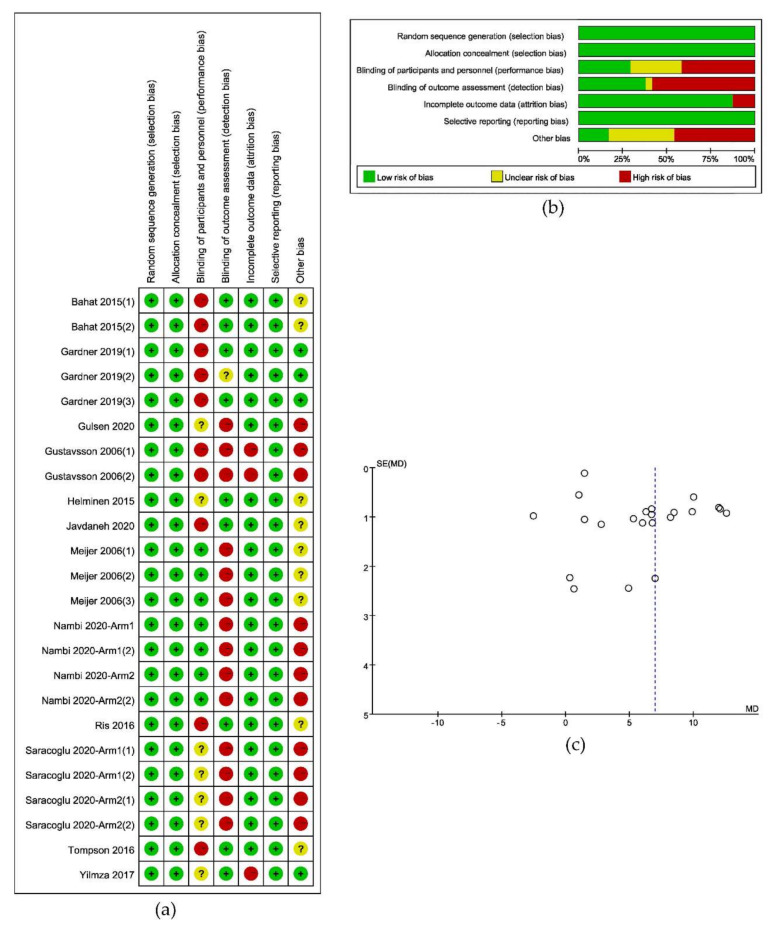
Results of risk of bias analysis: (**a**) Risk of bias summary; (**b**) Graph of risk of bias; (**c**) Funnel plot of included studies.

The result of the criteria for judging the risk of bias in the “Risk of bias assessment tool” would be shown in Figure 2a,b. A funnel plot of the included studies, as in Figure 2c, illustrated the risk of bias across studies and showed the relationship between the sample size and the effect. And the result of the assessment of evidence quality, which was made by following the GRADE, would be put into the Supplementary File (Figure S1).

### 3.3. Data Extraction and Management

**Figure 3:**
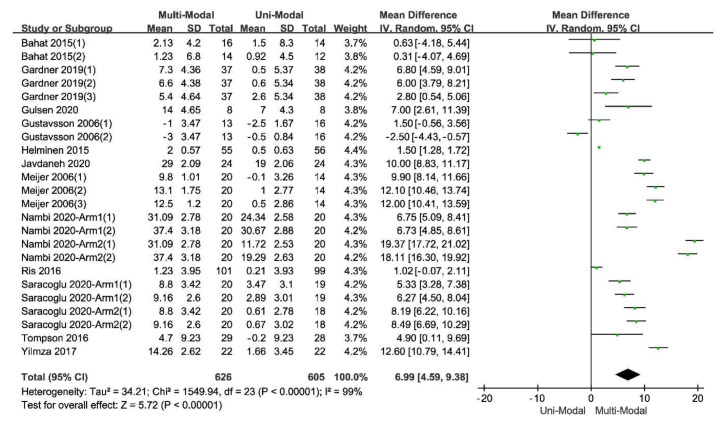
The forest plot of the comparison between the multi-modal therapies and uni-modal therapies.

In the comparison of multi-modal interventions and uni-modal interventions with the scores of TSK as the outcome, all the 12 included studies were divided into 24 independent trials. The result of original data processing was shown in Table 3, and the forest plots of the pooled effect, which were calculated by RevMan5.3, were shown in Figure 3.

The Q statistic of the meta-analysis of all included studies (equal to Chi^2^), T^2^, I^2^ and their 95% confidence intervals and prediction intervals of the comparisons’ effect could be seen in Table 4.

### 3.4. Additional Analysis

#### 3.4.1. Subgroup Analysis

The calculation of subgroup analysis was based on the randomized effect model by RevMan5.3. According to the result of the subgroup analysis, there was a large heterogeneity within studies in subgroup analysis since the I^2^ of each subgroup analysis was large, meaning that the different subgroups may not be the sources of the heterogeneity within the included studies, and it was necessary to do a meta-regression.

**Figure 4:**
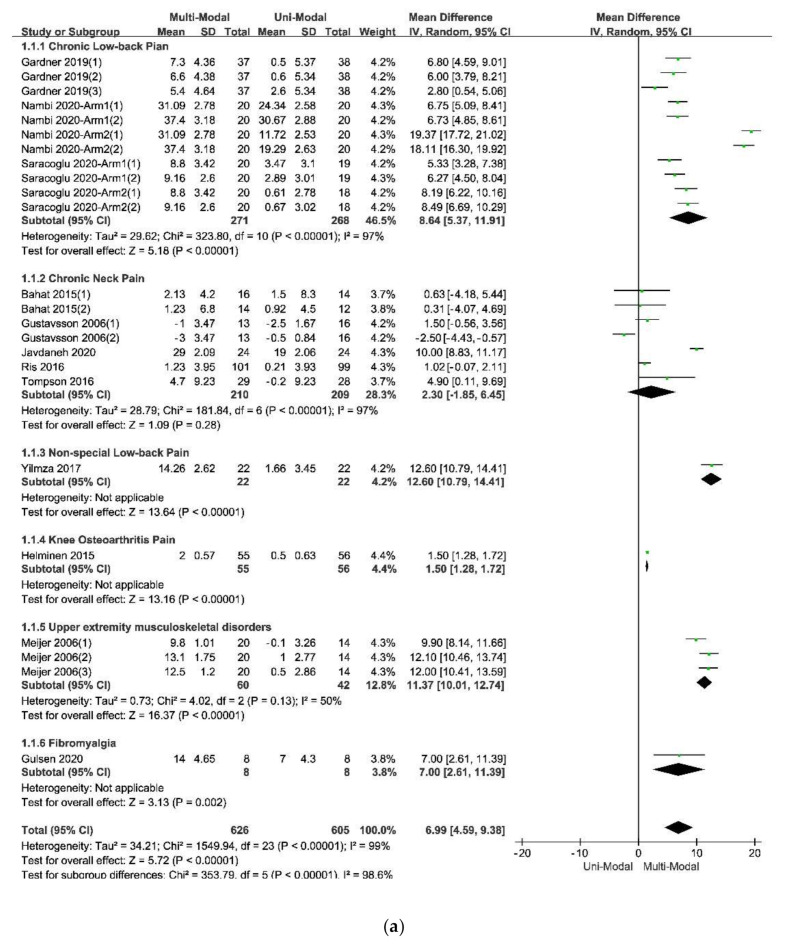

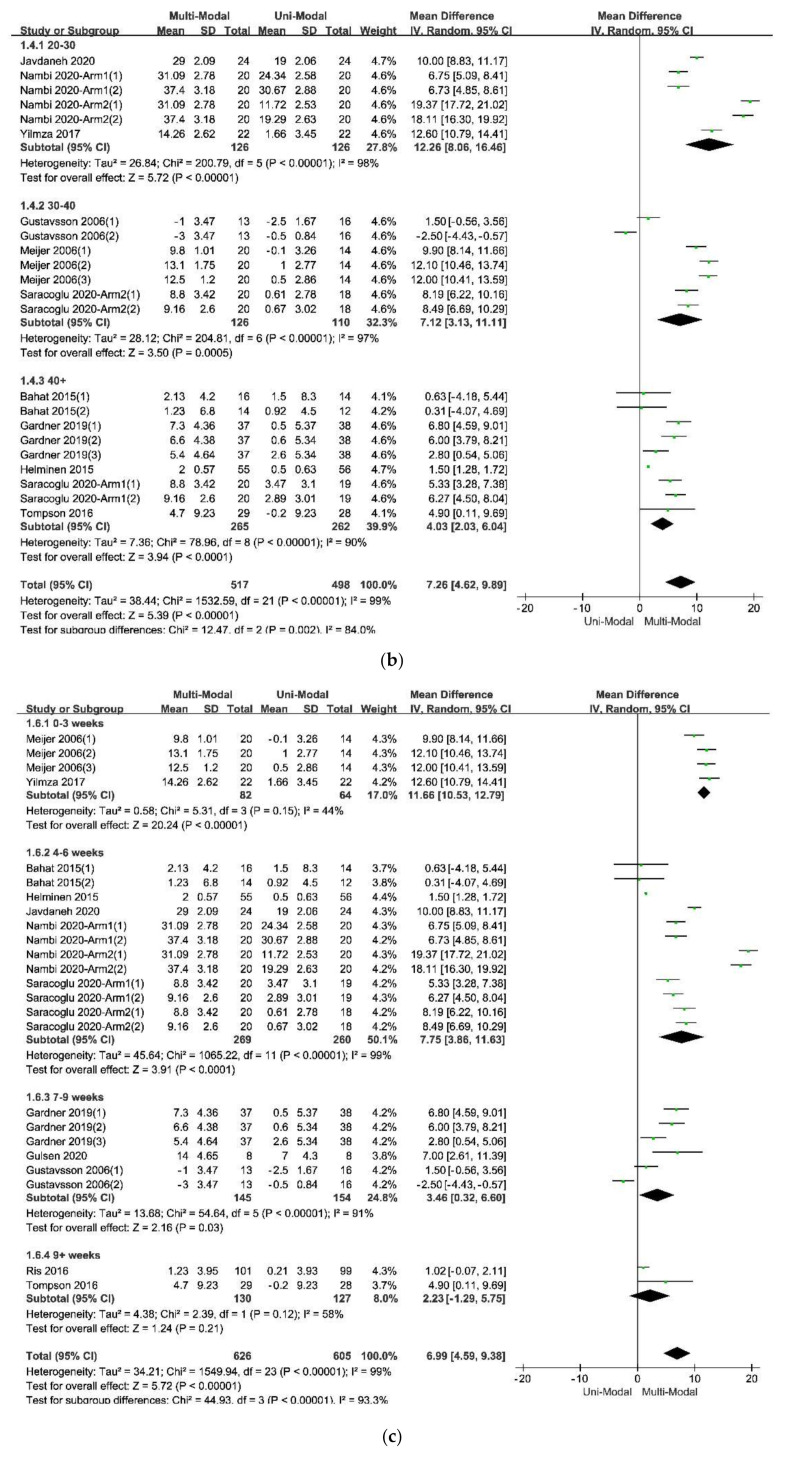

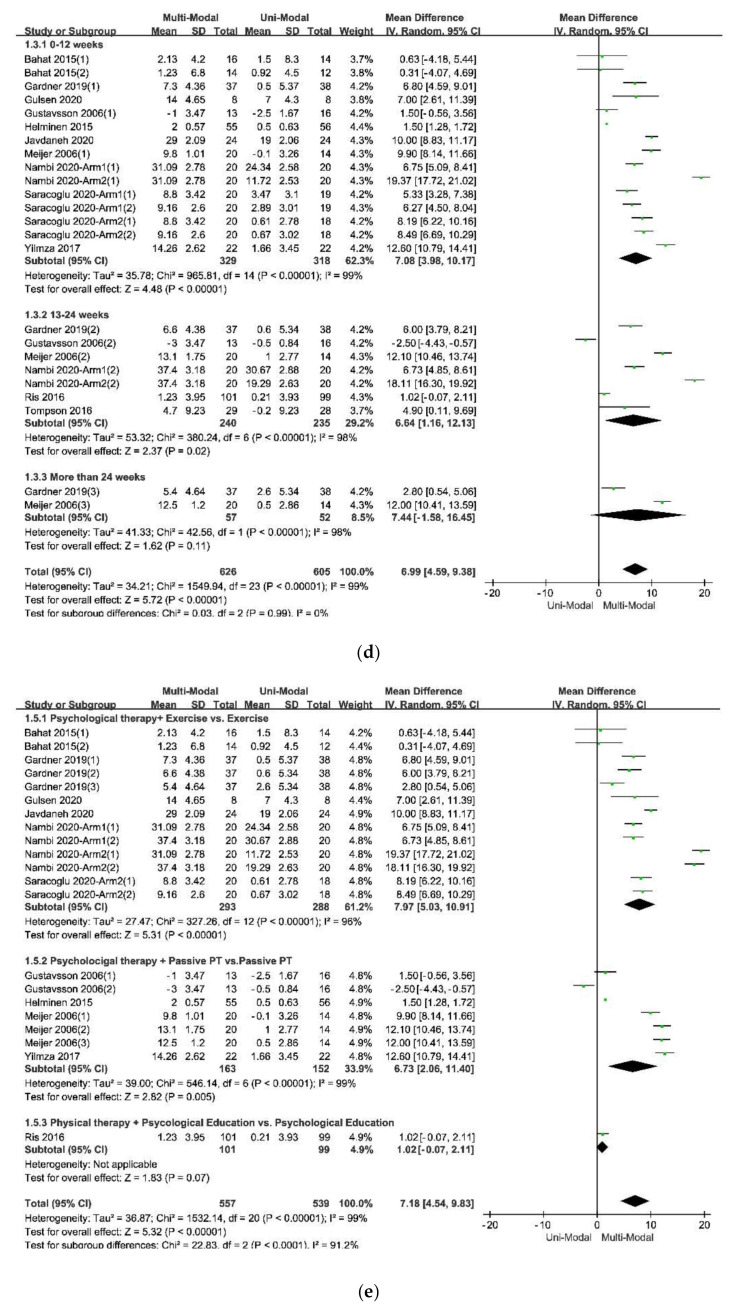
The forest plot of the subgroup analysis: (**a**) The subdivisions of different types of pain; (**b**) The subdivisions of different ranges of participants’ mean age; (**c**) The subdivisions of different durations of treatments; (**d**) The subdivisions of different follow-up times; (**e**) The subdivisions of different types of physical therapy in control groups.

According to the characteristics of participants and design of the included studies which might affect the effectiveness of therapies, there were five subdivisions in the subgroup analysis: (1) different types of pain, which included chronic low-back pain, chronic neck pain, and non-special low-back pain; (2) different ranges of participants’ mean age which included 20 to 30, 30 to 40, and more than 40 years old; (3) different durations of treatments which included 0 to 3 weeks, 4 to 6 weeks, 7 to 9 weeks and more than 9 weeks; (4) different follow-up times which included 0 to 12 weeks, 13 to 24 weeks and more than 24 weeks; (5) different types of uni-modal interventions in control groups, which include passive physical therapy, active exercise, and only psychological education (following the hypothesis that there were different effects between passive physical therapies, active exercise, and psychological-only interventions). The indexes of heterogeneity and their 95% confidence intervals were shown in Table 5, and all the forest plots of the subdivisions would be shown in Figure 4.

#### 3.4.2. Meta-Regression

**Figure 5:**
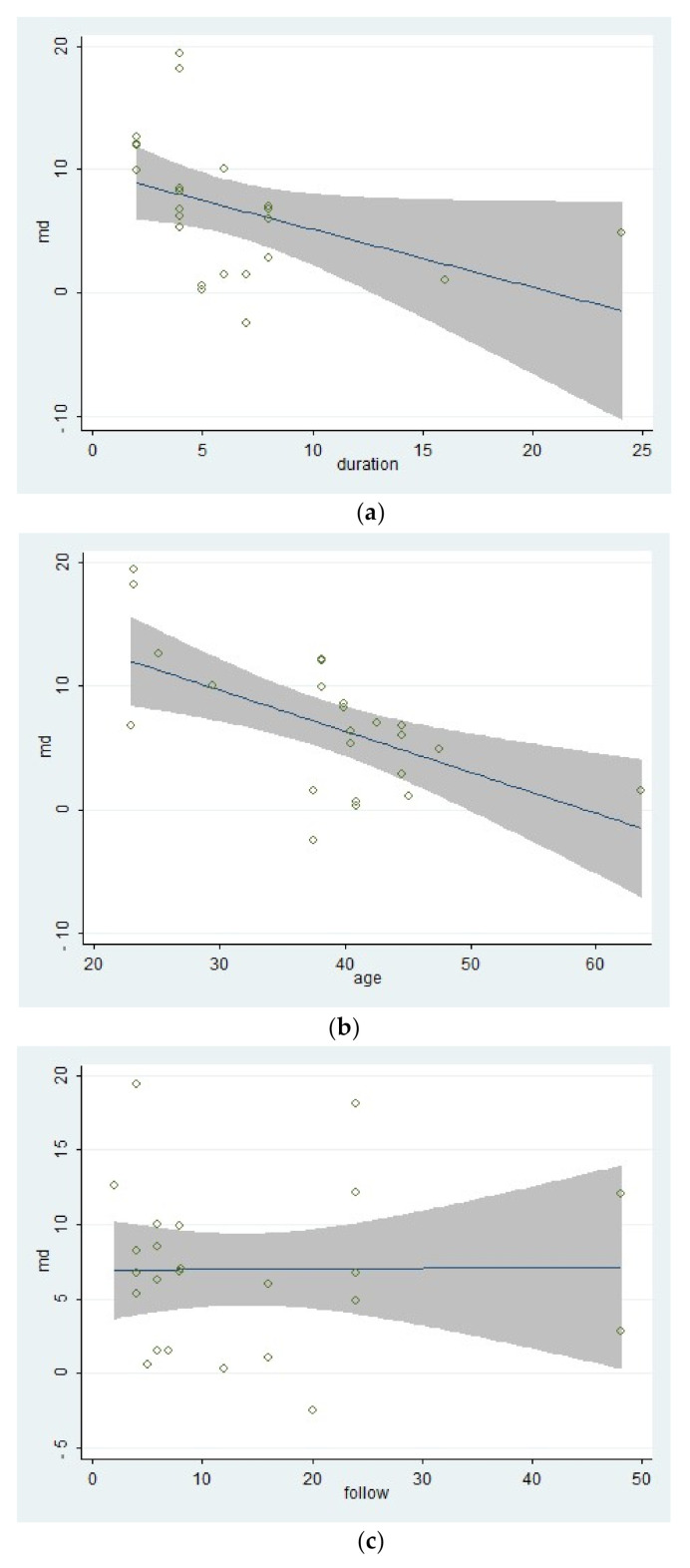
The bubble chats of the results of the three covariates in the meta-regression: (**a**) The duration of treatments; (**b**) Mean age of participants; (**c**) Follow-up times.

The large heterogeneity within the included studies made it necessary to do a meta-regression. The meta-regression made using STATA^®^ 12 (StataCorp LLC, College Station, TX, USA), and the results can be seen in Figure 5 and Table 6.

The result of the meta-regression calculation for the covariate “follow-up times” showed that the proportion of the residual variation due to heterogeneity, which could be represented by the statistic “I-squared_res”, was 99.58%. It meant that only 0.42% of the residual variation could be explained by between-study variance. And the result of the meta-regression calculation of the covariate “mean age of participants” showed that the proportion of the residual variation due to heterogeneity, which could be represented by the statistic “I^2^_residual_” (I-squared_res), was 96.11%. It meant that only 3.89% of the residual variation could be explained by between-study variance. The proportion of the heterogeneity could be explained by between-study variance, which could be represented by the statistic “adjusted R^2^” (Adj R-squared), which was 33.10%. The result of the meta-regression calculation of the covariate “duration of treatments” showed that the proportion of the residual variation due to heterogeneity, which could be represented by the statistic “I^2^_residual_” (I-squared_res), was 98.38%. It meant that only 1.62% of the residual variation could be explained by between-study variance. The proportion of the heterogeneity could be explained by between-study variance, which could be represented by the statistic “adjusted R^2^” (Adj R-squared), which was 14.46%. Lastly, the result of the Monte Carlo Permutation Test for Single Covariate of Meta-regression, the adjusted p-value changed from 0.139 to 0.008 to 1000 within the covariate “duration of treatments”, “mean age of participants”, and “follow-up times”, indicating that there might not be the type I error existing within the included studies. The bubble chats of the results of the three covariates in the meta-regression.

#### 3.4.3. Statistical Power

According to the result shown in Table 7, the meta-analysis’s statistical power was higher than any included study. Moreover, the statistical power of the heterogeneity analysis was less than that of the pooled effect because of the interactions between the covariables. The result was consistent with the previous hypothesis.

## 4. Discussion

In this review, the fear-avoidance model of pain was used to explain the fear of physical movement following musculoskeletal disorders, and the clinic term “Kinesiophobia” was used to define and describe fear in relation to pain. Kinesiophobia could be acquired through personal experience or social learning and could be measured by the Tampa Scale of Kinesiophobia (TSK). Studies used the scores of TSK-17 as one of the outcomes and compared therapies combined multi-modal from both psychological and physical perspectives with therapies in uni-modal were included in this review to summarize the evidence that might support the application of multi-modal therapies for musculoskeletal disorders and the following pain.

Although a considerable heterogeneity within the included studies, the pooled effect was positive with a statistical significance, indicating that multi-modal therapies had an advantage over uni-modal therapies. High-quality evidence reported that a long-lasting multi-modal program was superior to the exercise program in reducing disability, fear-avoidance beliefs and pain, and enhancing the quality of life of patients with different kinds of pain [15]. The effects were clinically tangible and lasted for at least one year after the intervention ended [15,20,22].

The results of the subgroup analysis in the subdivision of different types of pain, which was showed in Figure 4a indicated that the multi-modal therapies were more used in the treatments for chronic pain in the people’s trunk, especially in the neck and low back. This result was consistent with the previous fear-avoidance model about the fear of pain, which was that the experience of chronic, ongoing pain tends to become fear of pain [6,8]. What’s more, multi-modal therapies combined with physical therapies and psychological therapies had an advantage over therapies from a physical perspective, no matter the physical therapy was passive or active, as was showed in Figure 4e. Therefore, it was necessary to add psychological therapies in the treatments of chronic pain. A similar effect was found in studies that compared passive and active treatments for neck-shoulder pain and used the Visual Pain Scale (VAS) as an outcome measure [44]. Simultaneously, the age of participants, the duration of treatments, and the different follow-up times might affect the results. Within these factors, the participants’ age was more likely to be taken into consideration since the pooled effects showed a decreasing trend with the increase of age in Figure 4b. According to the previous study results, older people were more often had a pain of longer duration, more frequently and of more complexity, felt more disabled, received more pain treatments and had more health problems, and often used passive coping for pain [45]. The influence of different durations of treatments seemed unclear, as was in Figure 4c. Perhaps there were few studies comparing different durations of treatments for pain or kinesiophobia, and each treatment protocol had a different optimal duration. It might result in low homogeneity among studies and poor goodness of fit of regression equations, as shown in Table 6. At last, the pooled effects at different follow-up times seemed stale, as was in Figure 4d, indicating that the effects of multi-modal therapies might clinically tangible and lasted for a long time [15,46].

According to the meta-regression results, the covariate “follow-up times” might not be the source of the heterogeneity because that different follow-up times of included studies could hardly explain the residual variation due to between-study variance [29]. On the contrary, the differences of mean age of participants and the duration of treatments could explain part of the between-study variance, meaning that the two covariates might be part of the sources of the heterogeneity and would affect the effects of therapies. What’s more, the meta-regression of the mean age of participants had a significant statistical difference, showing that the effect of multi-modal therapies might decrease with age. This result might be related to the mental health and capacity of recovery of older adults [47,48]. Besides, the result of the meta-regression of the duration of treatments tended to be statistically significant. It indicated that there might be no additional benefit from increasing the duration of therapy for kinesiophobia. Finally, the goodness of fit of the model used in the meta-regression for these covariates was low, indicating that the results should be interpreted carefully.

A considerable heterogeneity within the included studies could be seen in the heterogeneity test in the meta-analysis and the subgroup analysis. The heterogeneity might come from the different designs of these studies. For example, the included studies had differences in the FITT characteristics (frequency, intensity, time, environments, and types) of the training plan [49,50]. Moreover, the different populations of the participants, the different blinding method, and some other factors, especially the different validities and reliability of the Tampa Scale of Kinesiophobia for participants with different educational backgrounds, culture, personalities, and types of musculoskeletal disorders [26,27,51], might lead a heterogeneity within studies.

This review had some limitations. Firstly, few studies reported the detailed pain duration of the participants or discussed the different effects between gender, leading it infeasible to make subgroup analysis or meta-regression for these covariates. Secondly, the statistical part of some studies did not consider the test-retest reliability of the Tampa Scale of Kinesiophobia, setting the test-retest reliability as 1.00 in their analysis of variance, which was impossible in a subjective questionnaire, so that the accuracy of their results was affected. Thirdly, in the search strategy, there might be an absence of data because the scores of the Tampa Scale of Kinesiophobia are usually reported as the secondary outcome. Finally, some studies didn’t use the Tampa Scale of Kinesiophobia to measure the fear of physical movements.

The risk of bias was supposed to exist, and the source is various. For example, there were many musculoskeletal disorders that could lead to a fear of physical movements. Still, not all studies in the field of physical rehabilitation reported the score of the Tampa Scale of Kinesiophobia. In fact, to all kinds of musculoskeletal disorders with the following pain, the fear of physical movements was very common [1]. What’s more, different shortened versions of the Tampa Scale of Kinesiophobia, such as TSK-13 and TSK-11, were used in other studies [52,53], making these studies could not be included in the review. Lastly, other resources of publication bias could not be excluded [54,55,56,57].

The statistical power of all pooled effect analysis in this review was larger than that in any single primary study, subgroup analysis, and the heterogeneity test. This result accords with the statistical law of meta-analysis [29].

## 5. Conclusions

It could be concluded that (1) Although a large heterogeneity within the included studies existed, the multi-modal therapies had advantages over uni-modal therapies for kinesiophobia caused by musculoskeletal disorders with a medium effect size; (2) Multi-modal therapies were more used in the treatments for chronic pain in the people’s trunk, especially in the neck and low back, and had an advantage over therapies from a physical perspective no matter the physical therapy was passive or active; (3) The effect of multi-modal therapies had a decreasing trend with the increase of age; (4) The influence of different durations of treatments seemed unclear; (5) The effects of multi-modal therapies might clinically tangible and lasted for a long time; (6) This review’s statistical methods, which considered the test-retest reliability when combing psychological measurements data that usually output as a secondary outcome, were mathematically feasible.

Further researchers should pay more attention to rehabilitation from psychophysiology perspectives when dealing with musculoskeletal disorders. It was suggested to take the fear of physical movements as one of the main treatment targets and regard the improvement of pain-related indicators as one of the primary assessment criteria of treatment effectiveness. It might be necessary to provide standards and official guides of therapies for fear of pain following musculoskeletal disorders. For example, therapists could benefit from more data on the reliability and validity of the Tampa Scale of Kinesiphobia for different types of musculoskeletal disorders.

## Figures and Tables

**Table 1:** The result of data collection.

Study	Study Design								Characteristics of Participants	
	Version of TSK		Multi-Modal Therapy Group			Uni-Modal Therapy Group			Mean Age	Type of Pain
	Language	R	Intervention	Duration of Intervention (Weeks)	N	Intervention	Duration of Intervention (Weeks)	N		
Gardner 2019 [34]	English	0.820	Education + Patient-led goal setting intervention	8	37	Standardised advice to exercise with a plan	8	38	44.51	Chronic Low-back Pian
Nambi 2020-Arm1 [43]	English	0.820	Virtual reality training	4	20	Isokinetic training with a plan	4	20	23.00	Chronic Low-back Pian
Nambi 2020-Arm2	English	0.820	Virtual reality training	4	20	Conventional training with a plan	4	20	23.25	Chronic Low-back Pian
Saracoglu 2020-Arm1 [40]	Turkish	0.806	Manual therapy + Supervised home exercise + Pain neuroscience education	4	20	Manual therapy + Supervised home exercise	4	19	40.50	Chronic Low-back Pian
Saracoglu 2020-Arm2	Turkish	0.806	Manual therapy + Supervised home exercise + Pain neuroscience education	4	20	Supervised home exercise	4	18	39.94	Chronic Low-back Pian
Gustavsson 2006 [32]	Swedish	0.910	Relaxation treatment	7	13	Treatment as usual	20	16	37.48	Chronic Neck Pain
Javdaneh 2020 [42]	Persian	0.920	Cognitive functional therapy + Scapular exercise	6	24	Scapular exercise with a plan	6	24	29.50	Chronic Neck Pain
Ris 2016 [36]	English	0.820	Physical training + Specific exercises + Pain education	16	101	Pain education	16	99	45.15	Chronic Neck Pain
Bahat 2015 [33]	Dutch	0.780	Cervical kinematic training + Interactive Virtual Reality training	5	16	Cervical kinematic training with a plan	5	16	40.88	Chronic Neck Pain
Tompson 2016 [37]	English	0.820	Cognitive-behavioural physiotherapy + Progressive neck exercise	24	29	Progressive neck exercise	24	28	47.53	Chronic Neck Pain
Yilmza 2017 [41]	Turkish	0.806	Virtual walking therapy	2	22	Traditional Physiotherapy	2	22	25.14	Non-special Low-back Pain
Helminen 2015 [39]	Finnish	0.890	Cognitive-behavioral group intervention contains skill training plan	6	55	Ordinary general practitioner care	6	56	63.64	Knee Osteoarthritis Pain
Meijer 2006 [35]	Dutch	0.780	Treatment combined physical and psychological sessions	2	20	Usual care by occupational health services	2	14	38.14	Upper extremity musculoskeletal disorders
Gulsen 2020 [38]	Turkish	0.806	Immersive Virtual Reality + Exercise	8	8	Exercise with a plan	8	8	42.50	Fibromyalgia
**Total**					**405**			**398**		
**Average**		**0.829**		**7.1**			**8.1**		**38.7**	

**Table 2:** The converted scores of TSK by the form of MEAN (SD).

Study	Group	Baseline (T0)		Follow-Up Times (T1)			Follow-Up Times (T2)			Follow-Up Times (T3)		
		TSK ^+^	N_0_	TSK ^+^	Duration	N_1_	TSK ^+^	Duration	N_2_	TSK ^+^	Duration	N_3_
Gardner 2019 [34]	OG	36.60 (7.50)	37	29.30 (6.90)	8	37	30.00 (5.30)	16	37	31.20 (7.90)	48	37
	CG	39.90 (9.30)	38	39.40 (8.30)	8	38	39.30 (8.10)	16	38	37.30 (8.00)	48	38
Nambi 2020-Arm1 [43]	OG	57.52 (4.80)	20	26.43 (3.50)	4	20	20.12 (2.50)	24	19			
	CG	58.11 (4.50)	20	27.54 (3.80)	4	20	21.21 (2.40)	24	20			
Nambi 2020-Arm2	OG	57.52 (4.80)	20	26.43 (3.50)	4	20	20.12 (2.50)	24	19			
	CG	57.93 (4.30)	20	46.21 (4.10)	4	20	38.64 (3.90)	24	19			
Saracoglu 2020-Arm1 [40]	OG	44.35 (4.30)	20	35.55 (5.75)	4	20	35.19 (3.99)	6				
	CG	45.10 (4.45)	19	41.63 (5.23)	4	19	42.21 (5.04)	6				
Saracoglu 2020-Arm2	OG	44.35 (4.30)	20	35.55 (5.75)	4	20	35.19 (3.99)	6				
	CG	45.55 (4.10)	18	44.94 (4.70)	4	18	44.88 (5.10)	6				
Gustavsson 2006 [32]	OG	26.00 (7.46)	13	27.00 (5.22)	7	13	29.00 (5.22)	20	13			
	CG	29.50 (1.67)	16	32.00 (0.00)	7	16	30.00 (2.00)	20	16			
Javdaneh 2020 [42]	OG	50.00 (5.23)	24	21.00 (5.22)	6	24						
	CG	49.00 (4.78)	24	30.00 (3.55)	6	24						
Ris 2016 [36]	OG	37.80 (0.69)	101	36.57 (4.50)	16	101						
	CG	37.70 (0.71)	99	37.49 (4.50)	16	99						
Bahat 2015 [33]	OG	32.75 (6.80)	16	30.13 (5.70)	5	16	31.23 (6.50)	12	14			
	CG	30.38 (5.80)	16	28.64 (9.90)	5	14	30.00 (5.90)	12	12			
Tompson 2016 [37]	OG	36.70 (7.10)	29	32.00 (14.11)	24	29						
	CG	33.60 (9.00)	28	33.80 (15.04)	24	28						
Yilmza 2017 [41]	OG	43.72 (4.32)	22	29.56 (4.04)	2	22						
	CG	40.36 (5.61)	22	38.70 (5.44)	2	22						
Helminen 2015 [39]	OG	35.00 (1.25)	55	33.00 (1.05)	6	55						
	CG	33.30 (1.35)	56	32.50 (1.33)	6	56						
Meijer 2006 [35]	OG	38.90 (1.51)	20	29.10 (1.54)	8	20	25.80 (2.65)	24	20	26.40 (1.90)	48	20
	CG	40.91 (1.81)	14	41.00 (1.68)	8	14	39.90 (3.16)	24	14	40.40 (2.65)	48	14
Gulsen 2020 [38]	OG	49.00 (4.44)	8	35.00 (7.41)	8	8						
	CG	47.00 (7.22)	8	40.00 (5.37)	8	8						

**^+^**: Mean (SD); TSK: Tampa scale of Kinesiophobia; OG: Operate group (Multi-modal therapy); CG: Control group (Uni-modal therapy).

**Table 3:** The result of original data processing.

Study	Duration of Intervention (Weeks)	Follow-Up Times (Weeks)	Mean Age	Experiment			Control		
				MD	SD	N	MD	SD	N
Gardner 2019 (1) [34]	8	8	44.51	7.30	4.36	37	0.50	5.37	38
Gardner 2019 (2)	8	16	44.51	6.60	4.38	37	0.60	5.34	38
Gardner 2019 (3)	8	48	44.51	5.40	4.64	37	2.60	5.34	38
Nambi 2020-Arm1 (1) [43]	4	4	23.00	31.09	2.78	20	24.34	2.58	20
Nambi 2020-Arm1 (2)	4	24	23.00	37.40	3.18	20	30.67	2.88	20
Nambi 2020-Arm2 (1)	4	4	23.25	31.09	2.78	20	11.72	2.53	20
Nambi 2020-Arm2 (2)	4	24	23.25	37.40	3.18	20	19.29	2.63	20
Saracoglu 2020-Arm1 (1) [40]	4	4	40.50	8.80	3.42	20	3.47	3.10	19
Saracoglu 2020-Arm1 (2)	4	6	40.50	9.16	2.60	20	2.89	3.01	19
Saracoglu 2020-Arm2 (1)	4	4	39.94	8.80	3.42	20	0.61	2.78	18
Saracoglu 2020-Arm2 (2)	4	6	39.94	9.16	2.60	20	0.67	3.02	18
Gustavsson 2006 (1) [32]	7	7	37.48	-1.00	3.47	13	-2.50	1.67	16
Gustavsson 2006 (2)	7	20	37.48	-3.00	3.47	13	-0.50	0.84	16
Javdaneh 2020 [42]	6	6	29.50	29.00	2.09	24	19.00	2.06	24
Ris 2016 [36]	16	16	45.15	1.23	3.95	101	0.21	3.93	99
Bahat 2015 (1) [33]	5	5	40.88	2.13	4.20	16	1.50	8.30	14
Bahat 2015 (2)	5	12	40.88	1.23	6.80	14	0.92	4.50	12
Tompson 2016 [37]	24	24	47.53	4.70	9.23	29	-0.20	9.23	28
Yilmza 2017 [41]	2	2	25.14	14.26	2.62	22	1.66	3.45	22
Helminen 2015 [39]	6	6	63.64	2.00	0.57	55	0.50	0.63	56
Meijer 2006 (1) [35]	2	8	38.14	9.80	1.01	20	-0.10	3.26	14
Meijer 2006 (2)	2	24	38.14	13.10	1.75	20	1.00	2.77	14
Meijer 2006 (3)	2	48	38.14	12.50	1.20	20	0.50	2.86	14
Gulsen 2020 [38]	8	8	42.50	14.00	4.65	8	7.00	4.30	8

MD: Mean Difference; SD: Standard Deviation; N: Sample Size.

**Table 4:** The result of heterogeneity test of all included studies.

Statistics of Heterogeneity Test				95% CI of T^2^		95% CI of I^2^		Prediction Intervals	
df	Q	I^2^	Tau^2^	LL	UL	LL	UL	LL	UL
23	1549.94	99%	34.21	3.37	4.09	0.87	0.89	0.00 *	19.33

df: Degree of freedom; LL: Lower limit; UL: Upper limit; *: the true value is less than 0.

**Table 5:** The result of heterogeneity test of subgroup analysis.

Outcome Subdivision	Subgroups	Statistics of Heterogeneity Test				95% CI of T^2^		95% CI of I^2^		Prediction Intervals	
		df	Q	I^2^	Tau^2^	LL	UL	LL	UL	LL	UL
**Types of Pain**	CLBP	10	323.8	97%	29.62	3.63	5.37	79%	85%	0.00 *	21.29
	CNP	6	181.84	97%	28.79	3.39	5.70	78%	85%	0.00 *	16.59
	UEMD	2	4.02	50%	0.73	0 *	1.18	0% *	62%	0.00 *	26.55
**Mean Age of Participants**	20–30	5	49.24	90%	2.71	0.39	1.02	56%	77%	6.14	18.38
	30–40	6	127.78	95%	5.34	0.70	1.28	73%	83%	0.14	14.10
	40+	8	63.73	87%	0.61	0.10	0.24	53%	73%	1.01	7.05
**Duration of Treatments**	0–3 weeks	3	5.31	44%	0.58	0 *	0.98	0% *	57%	7.03	16.29
	4–6 weeks	11	1065.22	99%	45.64	3.73	4.74	89%	91%	0.00 *	23.12
	7–9 weeks	5	54.64	91%	13.68	1.95	4.86	59%	78%	0.00 *	14.31
	9+ weeks	1	2.39	58%	4.38	0 *	6.85	0%	69%	N/N	N/N
**Follow-up Times**	0–12 weeks	14	965.81	99%	35.78	3.40	4.34	87%	89%	0.00*	20.28
	13–24 weeks	6	380.24	98%	53.32	4.90	7.19	85%	89%	0.00 *	25.90
	24+ weeks	1	37.07	97%	13.05	1.02	3.15	74%	90%	N/N	N/N
**Physical Therapy in Control Groups**	Active Exercise	12	327.26	96%	27.47	3.63	5.34	78%	84%	0.00 *	19.82
	Passive Therapy	6	546.14	99%	39.00	3.13	4.36	88%	91%	0.00 *	23.27

df: Degree of freedom; LL: Lower limit; UL: Upper limit; *: the true value is less than 0; CNP: Chronic neck pain; CLBP: Chronic low-back pain; UEMD: Upper extremity musculoskeletal disorders; N/N: when df = 1, the t-value could not be calculated so that the prediction intervals could not be estimated as well.

**Table 6:** Meta-regression result: The follow-up times and the SMD of effect.

Meta-Regression				
Results		Covariate		
Item	Index	Duration of Treatments	Mean Age of Participants	Follow-Up Times (Week)
Number of obs	N	24	24	24
REML estimate of between-study variance	Tau^2^	24.39	19.08	29.86
% residual variation due to heterogeneity	I-squared_res	98.38%	96.11%	99.58%
Proportion of between-study variance explained	Adj R-squared	14.46%	33.10%	−4.74%
Statistical Significance	*P*-value	0.052	0.002	0.963
Monte Carlo Permutation Test	Adjusted *P*-value	0.139	0.008	1.000

**Table 7:** The result of statistical power test of all included studies and meta-analysis.

Study	Statistical Power of Study	Subdivisions	Subgroups	Statistical Power of Effect Combination	Statistical Power of Heterogeneity Test
Gardner 2019 (1) [34]	13.45%	Types of Pain	CLBP	100.00%	51.76%
Gardner 2019 (2)	18.34%		CNP	48.92%	39.09%
Gardner 2019 (3)	8.15%		UEMD	100.00%	22.36%
Nambi 2020-Arm1 (1) [43]	5.62%	Follow-up times	0–12 weeks	100.00%	61.90%
Nambi 2020-Arm1 (2)	11.75%		13–24 weeks	99.66%	39.09%
Nambi 2020-Arm2 (1)	17.54%		24+ weeks	48.16%	16.58%
Nambi 2020-Arm2 (2)	5.82%	Mean Age of Participants	20–30	100.00%	35.41%
Saracoglu 2020-Arm1 (1) [40]	14.54%		30–40	99.99%	39.09%
Saracoglu 2020-Arm1 (2)	6.13%		40+	99.42%	45.79%
Saracoglu 2020-Arm2 (1)	5.61%	Physical Therapy in Control Groups	Active Exercise	100.00%	57.10%
Saracoglu 2020-Arm2 (2)	21.80%		Passive Therapy	100.00%	39.09%
Gustavsson 2006 (1) [32]	19.81%	Duration of Treatments	0–3 weeks	100.00%	27.16%
Gustavsson 2006 (2)	11.61%		4–6 weeks	100.00%	54.51%
Javdaneh 2020 [42]	56.06%		7–9 weeks	55.56%	35.41%
Ris 2016 [36]	64.57%		9+ weeks	14.78%	16.58%
Bahat 2015 (1) [33]	67.83%	Total		100.00%	77.95%
Bahat 2015 (2)	9.84%				
Tompson 2016 [37]	10.11%				
Yilmza 2017 [41]	7.36%				
Helminen 2015 [39]	9.19%				
Meijer 2006 (1) [35]	23.34%				
Meijer 2006 (2)	11.27%				
Meijer 2006 (3)	52.40%				
Gulsen 2020 [38]	27.89%				

CNP: Chronic neck pain; CLBP: Chronic low-back pain; UEMD: Upper extremity musculoskeletal disorders.

## Supplementary Materials

The following are available online at https://www.mdpi.com/1660-4601/17/24/9439/s1, Figure S1: The result of the GRADE assessment of evidence quality.
