# Supplementary material for: Effect of Multi-Modal Therapies for Kinesiophobia Caused by Musculoskeletal Disorders: A Systematic Review and Meta-Analysis
Source: Int J Environ Res Public Health. 2020 Dec 16;17(24):9439. doi: 10.3390/ijerph17249439 (PMC7766030; doi:10.3390/ijerph17249439)
Supplement: Supplementary file 1 [file ijerph-17-09439-s001.pdf]

Author(s):
Date: 2020-11-09
Question: Multi-disciplinary therapies vs Single-disciplinary therapies for kinesiphobia caused by musculoskeletal injuries
Settings:
Bibliography: . Multi-discislinary therapies for kinesiphobia caused by musculoskeletal injuries. Cochrane Database of Systematic Reviews [Year], Issue [Issue].

Quality assessment							Summary of findings					
							No of patients		Effect		Quality	Importance
No of studies	Design	Limitations	Inconsistency	Indirectness	Imprecision	Other considerations	Multi-disciplinary therapies	Single-disciplinary therapies	Relative (95% CI)	Absolute		
**TSK-17(Pain) - Chronic Low-back Pian (follow-up mean 16.7 weeks; measured with: Tampa Scale of Kinesiophobia; range of scores: 17-68; Better indicated by lower values)**												
11	randomised trials	no serious limitations	serious[1]	no serious indirectness	no serious imprecision	reporting bias[1]	271	268	-	MD 8.64 higher (5.37 to 11.91 higher)	⊕⊕OO LOW	IMPORTANT
**TSK-17(Pain) - Chronic Neck Pain (follow-up mean 12.9 weeks; measured with: Tampa Scale of Kinesiophobia; range of scores: 17-68; Better indicated by lower values)**												
7	randomised trials	no serious limitations	serious[1]	no serious indirectness	no serious imprecision	reporting bias[1]	210	209	-	MD 2.3 higher (1.85 lower to 6.45 higher)	⊕⊕OO LOW	IMPORTANT
**TSK-17(Pain) - Non-special Low-back Pain (follow-up mean 2 weeks; measured with: Tampa Scale of Kinesiophobia; range of scores: 17-68; Better indicated by lower values)**												
1	randomised trials	no serious limitations	no serious inconsistency[1]	no serious indirectness	no serious imprecision	reporting bias[1]	22	22	-	MD 12.6 higher (10.79 to 14.41 higher)	⊕⊕OO LOW	IMPORTANT
**TSK-17(Pain) - Knee Osteoarthritis Pain (follow-up mean 6 weeks; measured with: Tampa Scale of Kinesiophobia; range of scores: 17-68; Better indicated by lower values)**												
1	randomised trials	no serious limitations	no serious inconsistency[1]	no serious indirectness	no serious imprecision	reporting bias[1]	55	56	-	MD 1.5 higher (1.28 to 1.72 higher)	⊕⊕OO LOW	IMPORTANT
**TSK-17(Pain) - Upper extremity musculoskeletal disorders (follow-up mean 26.7 weeks; measured with: Tampa Scale of Kinesiophobia; range of scores: 17-68; Better indicated by lower values)**												
3	randomised trials	no serious limitations	serious[1]	no serious indirectness	no serious imprecision	reporting bias[1]	60	42	-	MD 11.37 higher (10.01 to 12.74 higher)	⊕⊕OO LOW	IMPORTANT
**TSK-17(Pain) - Fibromyalgia (follow-up mean 8 weeks; measured with: Tampa Scale of Kinesiophobia; range of scores: 17-68; Better indicated by lower values)**												
1	randomised trials	no serious limitations	no serious inconsistency[1]	no serious indirectness	no serious imprecision	reporting bias[1]	8	8	-	MD 7 higher (2.61 to 11.39 higher)	⊕⊕OO LOW	IMPORTANT
**TSK-17(Total) (follow-up mean 15.1 weeks; measured with: Tampa Scale of Kinesiophobia; range of scores: 17-68; Better indicated by lower values)**												
24	randomised trials	no serious limitations[1]	serious[1]	no serious indirectness	no serious imprecision	reporting bias[1]	626	605	-	MD 6.99 higher (4.59 to 9.38 higher)	⊕⊕OO LOW	CRITICAL
**TSK-17(Follow-up) - 0-12 weeks (follow-up mean 6 weeks; measured with: Tampa Scale of Kinesiophobia; range of scores: 17-68; Better indicated by lower values)**												
15	randomised trials	no serious limitations	serious[1]	no serious indirectness	no serious imprecision	reporting bias[1]	329	318	-	MD 7.08 higher (3.98 to 10.17 higher)	⊕⊕OO LOW	IMPORTANT
**TSK-17(Follow-up) - 13-24 weeks (follow-up mean 21.1 weeks; measured with: Tampa Scale of Kinesiophobia; range of scores: 17-68; Better indicated by lower values)**												
7	randomised trials	no serious limitations	serious[1]	no serious indirectness	no serious imprecision	reporting bias[1]	240	235	-	MD 6.64 higher (1.16 to 12.13 higher)	⊕⊕OO LOW	IMPORTANT
**TSK-17(Follow-up) - More than 24 weeks (follow-up mean 48 weeks; measured with: Tampa Scale of Kinesiophobia; range of scores: 17-68; Better indicated by lower values)**												
2	randomised trials	no serious limitations	serious[1]	no serious indirectness	no serious imprecision	reporting bias[1]	57	52	-	MD 7.44 higher (1.58 lower to 16.45 higher)	⊕OOO VERY LOW	IMPORTANT
**TSK-17(Mean Age) - 20-30 (follow-up mean 10.7 weeks; measured with: Tampa Scale of Kinesiophobia; range of scores: 17-68; Better indicated by lower values)**												
6	randomised trials	no serious limitations	serious[1]	no serious indirectness	no serious imprecision	reporting bias[1]	126	126	-	MD 12.26 higher (8.06 to 16.46 higher)	⊕⊕OO LOW	IMPORTANT
**TSK-17(Mean Age) - 30-40 (follow-up mean 16.7 weeks; measured with: Tampa Scale of Kinesiophobia; range of scores: 17-68; Better indicated by lower values)**												
7	randomised trials	no serious limitations	serious[1]	no serious indirectness	no serious imprecision	reporting bias[1]	126	110	-	MD 7.12 higher (3.13 to 11.11 higher)	⊕⊕OO LOW	IMPORTANT
**TSK-17(Mean Age) - 40+ (follow-up mean 11.7 weeks; measured with: Tampa Scale of Kinesiophobia; range of scores: 17-68; Better indicated by lower values)**												
9	randomised trials	no serious limitations	no serious inconsistency	no serious indirectness	no serious imprecision	reporting bias[1]	265	262	-	MD 4.03 higher (2.03 to 6.04 higher)	⊕⊕⊕O MODERATE	CRITICAL
**TSK-17(Intervention) - Psychological therapy+ Exercise vs. Exercise (follow-up mean 13 weeks; measured with: Tampa Scale of Kinesiophobia; range of scores: 17-68; Better indicated by lower values)**												
13	randomised trials	no serious limitations	serious[1]	no serious indirectness[1]	no serious imprecision	reporting bias[1]	293	288	-	MD 7.97 higher (5.03 to 10.91 higher)	⊕⊕OO LOW	CRITICAL
**TSK-17(Intervention) - Psycholocigal therapy + Passive PT vs.Passive PT (follow-up mean 16.4 weeks; measured with: Tampa Scale of Kinesiophobia; range of scores: 17-68; Better indicated by lower values)**												
7	randomised trials	no serious limitations	serious[1]	no serious indirectness	no serious imprecision	reporting bias[1]	163	152	-	MD 6.73 higher (2.06 to 11.4 higher)	⊕⊕OO LOW	IMPORTANT
**TSK-17(Intervention) - Physical therapy + Psycological Education vs. Psychological Education (follow-up mean 16 weeks; measured with: Tampa Scale of Kinesiophobia; range of scores: 17-68; Better indicated by lower values)**												
1	randomised trials	no serious limitations	no serious inconsistency	no serious indirectness	no serious imprecision	reporting bias[1]	101	99	-	MD 1.02 higher (0.07 lower to 2.11 higher)	⊕⊕OO LOW	IMPORTANT
**TSK-17(Intervention Duration) - 0-3 weeks (follow-up mean 20.5 weeks; measured with: Tampa Scale of Kinesiophobia; range of scores: 17-68; Better indicated by lower values)**												
4	randomised trials	no serious limitations	serious[1]	no serious indirectness	no serious imprecision	reporting bias[1]	82	64	-	MD 11.66 higher (10.53 to 12.79 higher)	⊕⊕OO LOW	NOT IMPORTANT
**TSK-17(Intervention Duration) - 4-6 weeks (follow-up mean 8.25 weeks; measured with: Tampa Scale of Kinesiophobia; range of scores: 17-68; Better indicated by lower values)**												
12	randomised trials	no serious limitations	serious[1]	no serious indirectness	no serious imprecision	reporting bias[1]	269	260	-	MD 7.75 higher (3.86 to 11.63 higher)	⊕⊕OO LOW	NOT IMPORTANT
**TSK-17(Intervention Duration) - 7-9 weeks (follow-up mean 17.8 weeks; measured with: Tampa Scale of Kinesiophobia; range of scores: 17-68; Better indicated by lower values)**												
6	randomised trials	no serious limitations	serious[1]	no serious indirectness	no serious imprecision	reporting bias[1]	145	154	-	MD 3.46 higher (0.32 to 6.6 higher)	⊕⊕OO LOW	NOT IMPORTANT
**TSK-17(Intervention Duration) - 9+ weeks (follow-up mean 20 weeks; measured with: Tampa Scale of Kinesiophobia; range of scores: 17-68; Better indicated by lower values)**												
2	randomised trials	no serious limitations	serious[1]	no serious indirectness	no serious imprecision	reporting bias[1]	130	127	-	MD 2.23 higher (1.29 lower to 5.75 higher)	⊕⊕OO LOW	NOT IMPORTANT

[1] No explanation was provided

Figure S1. The result of the GRADE assessment of evidence quality.
